# Towards a suicide-responsive police culture: police experiences of working with suicide

**DOI:** 10.1080/10439463.2024.2372684

**Published:** 2024-07-03

**Authors:** Michelle O’Reilly, Barney Thorne, Philippa Smith, Nikki Kiyimba

**Affiliations:** a University of Leicester and Leicestershire Partnership NHS Trust, University Road, Leicester, UK; b Head of Mental Health/Suicide Prevention Lead, Leicestershire Police, Wigston Police Station, Wigston, UK; c Public Protection and Vulnerability, British Transport Police HQ, London, UK; d Mātai Rongo Trauma-Responsive Psychological Services, ReDefined Wellbeing Hub, Bay of Plenty, Papamoa Beach, New Zealand

**Keywords:** Police, suicide, qualitative, trauma, training

## Abstract

Across the globe suicide rates are creating concern, and the World Health Organisation (WHO) argues that suicide is the responsibility of everyone. The WHO recognises that police have an important role in suicide prevention and the management of mental health crises. We suggest that police officers and staff are inevitably impacted by the emotional labour of meeting the demands of this aspect of the role. We argue for more research attention on the experiences and views of police who work with suicide. This study comprised qualitative reflective interviews with police in the UK, Guyana and New Zealand, with the focus on identifying commonalities in experience across the three locations. Four themes were generated through reflective thematic analysis: (1) the context; (2) the traumatic impact; (3) the difficult aspects of this work and (4) organisational opportunities for supporting police officers and staff. Our data illustrated that suicide is qualitatively different from other forms of death, and the nature of the work did have considerable emotional and traumatic impact on police. We conclude our paper with several recommendations for police forces to build their welfare support for staff. These recommendations could help promote a healthier workforce and recognise the specific issues associated with this part of the role.

## Introduction

Suicide is defined as an action directly or indirectly resulting in a ‘self-inflicted death’ (Ilgün *et al.* 2020), with figures showing that worldwide on average a person dies by suicide every 40s, with 20 times more than that attempting suicide (WHO 2014). Suicide prevention is therefore a global priority that requires a multisectoral approach tailored to local contexts, with suitable budget allocations (WHO 2018). A range of agencies play an important role in this, one of which is the police. Frontline police officers are frequently called to mental health emergencies including those in suicidal crisis (WHO 2009). When attending individuals in suicidal crisis, police have a direct role in suicide intervention at the scene (Sher 2016).

Police work in all areas of suicide prevention, intervention and postvention. Preventing suicides and reducing suicide rates are typically at the centre of a suicide prevention strategy (DoHSC 2023). Suicide intervention involves supporting persons in crisis (Samaritans n.d.), and postvention work involves facilitating the grieving process, mitigating the negative impact of exposure and preventing further suicide (Howard *et al.* 2022). Early responders like the police play a critical role in the experiences of those seeking support (Duval *et al.* 2023). Broadly speaking, police work includes supporting people in suicidal crisis, negotiating with those attempting suicide, attending the scene of sudden deaths, handling the bodies of those who have died by suicide, delivering the death message to family members and communicating with the coroner post-mortem. In representing all of these aspects of the police role throughout this paper, we utilise the phrase ‘working with suicide’.

Despite these first responder responsibilities, evidence suggests that police personnel rarely have specialist training in working with suicide (Marzano *et al.* 2016). Marzano *et al* argued that training could build confidence and knowledge and raise awareness of officers’ own mental health needs. Police officers tend to have high levels of mental health needs, as policing is a stressful occupation, often dealing with unpredictable events and police are frequently exposed to the trauma of others (Violanti *et al.* 2011). Many professional groups are cumulatively exposed to the trauma of suicide (Reeves 2021). Research has illustrated that an encounter with death is the strongest predictor of Post-Traumatic Stress Disorder (PTSD) for American police officers (Robinson *et al.* 1997).

Some occupational groups, including police have an elevated risk of suicide themselves because of occupational stress, exposure to traumatic situations, working shifts, unmanageable workloads, workplace bullying and relationship challenges (Chae and Boyle 2013; Waters *et al.* 2016). Occupational stress is exacerbated by the decline in the number of officers (Allen and Zayed 2019), which means there are less officers to undertake the level of work and thus requires organisations to be strategic in managing workloads (Clements *et al.* 2021). Additionally, the presence of PTSD, as well as attending a traumatic critical incident increases suicidal ideation in police officers (Violanti 2017). Consequently, officers are more likely to experience high levels of depression and alcohol dependency (Foley and Massey 2019), as well as suicidal ideation and suicide (Violanti *et al.* 2011, Violanti 2017). It is arguable that work with sudden death, and more specifically with suicidal crisis and death by suicide form a specific occupational burden for police officers, which problematically intersects with other stressful aspects of the occupation. This is especially important given that research indicates that police exposed to a high number of suicides in their career are at greater risk of taking their own lives (Cerel *et al.* 2019).

Given the potential consequences for sickness, depression, alcohol dependency, PTSD and suicidal ideation from working with people experiencing trauma, it is evident that more research is needed. Specifically, greater attention is necessary to explore what it is like for police officers and those in related police staff roles working with suicide. All professionals, whether working directly or indirectly with suicide have an important role to play, but consequently may need support, welfare checks or trauma treatment because of the impact on them. However, much of the literature has focused on wider stressors and challenges of the occupation of policing. There is less exploration of what police themselves feel, think, believe or experience with this specific aspect of their occupational role. In this paper, we focus on police officers and staff in three countries, the United Kingdom (UK), Guyana (GY) and New Zealand (NZ) which captures a broad range of policing experiences and cultural influences. Statistically, suicide rates are high in all three countries with global comparative figures collated in 2019 by the WHO, showing that Guyana has the second highest suicide rates in the world, in that year of 40.9 (per 100 k), and New Zealand had 10.3 (per 100 k) and the lowest of our included countries was the UK at 6.9 (per 100 k) (WHO 2020).

For the UK, Public Health England (PHE) (2020) produced an online suicide prevention atlas of suicide data for each Local Authority for action (Simms and Scowcroft 2018). This focused on real-time surveillance data, crisis management and support for those bereaved. This was an initiative predominantly led by the police (PHE 2020). In the UK, the police are a central agency for the collection of real-time surveillance data, supporting crisis management, referring those bereaved by suicide and in identifying suicide prevention strategy in local areas (Thorne and O’Reilly 2022). In this context, it is notable that in England the criteria for a conclusion of suicide changed from ‘beyond reasonable doubt’ to a ‘balance of probabilities’, which is arguably more accurate (Appleby *et al.* 2019). In Guyana, suicide rates are now ranked as the highest prevalence in the world, which may reflect their complex history and diverse ethnic and cultural composition (Shaw *et al*. 2022). Shaw *et al* concluded their systematic review by noting that there is insufficient research in Guyana related to suicide. This is especially true in relation to police work with suicide (see Johnson 2019 for one recent study). In New Zealand, suicide prevalence remains a problem, although data illustrate some changing trends in relation to age, with younger people more likely to die by suicide than older people (Snowdon 2017). The complexity of challenges faced in New Zealand include the pathologising of indigenous Māori practices as mental health problems (Shahtahmasebi and Cassidy 2014). This has implications for suicide prevention in relation to misinterpretations around medicalising mental health which can make service provision culturally inappropriate (Shahtahmasebi 2020). Additionally, in New Zealand, since 2016, mental health emergency calls to the police have increased by 60% and are projected to rise further by 2025 (New Zealand Police 2021). Internationally, calls for people in crisis have been managed by police, ambulance and mental health services working separately, but recently there have been explorations for a more coordinated multi-agency co-response. For example, co-response initiatives have been underway in Australia, the USA and Canada (Balfour *et al.* 2022, McKenna *et al.* 2015, Marcus and Stergiopoulos 2022), and more recently in New Zealand (Every-Palmer *et al.* 2023).

While it is almost impossible to disentangle work with suicide from the other traumatic and stressful elements of policing, it is necessary and valuable to focus on this part of the role to gain a better understanding of how working with suicide affects those in practice. While we acknowledge differences in the cultural, social and political landscape of each country which would inform differences in policing practices and experiences, our goal in this research project was to explore the similarities. We therefore asked the research question ‘what are the commonalities in experience of police officers and staff working with suicide in the three international locations?’.

## Method

We utilised a thematic qualitative design informed by practice. This project was co-developed by researchers and practitioners, as the two UK forces were motivated to explore what might be helpful in improving support for the workforce. Two of the authors [MO & NK] created the practice-driven research question and designed the co-research approach with partners (BT & PS). These partners engaged from design to data collection/analysis and dissemination. The applied nature of the qualitative co-research approach to blend practice-based ideologies necessitated a dialectical pluralist position to facilitate integration of ideas, stakeholders’ positionality and mapping of core findings (Johnson 2019). This was underpinned by a social constructionist framework that elevates the value of language in creating and agreeing meaning (Burr 2003).

## Data collection and recruitment

Semi-structured reflective interviews were conducted with 67 police officers and staff. In the UK, participants from the national British Transport Police (BTP) (representing England, N. Ireland, Scotland and Wales), and one local English regional force (*N* = 32) participated. Also, from BTP we recruited staff working in the Force Control Room (*N* = 11). From Guyana, we included officers from one regional force (*N* = 12) and from New Zealand, we included officers and staff from one regional force (*N* = 12). Participants were recruited via our partners in each country who volunteered to participate, and numbers were representative of the size of the force and access available through this sampling approach. In the UK, these partners had specialist roles in relation to suicide within the police forces, in Guyana, partners were members of a not-for-profit agency with local connections, and in New Zealand partners supported through a national research office. The interview schedule was designed to address the research agenda. This included views of operational issues of suicide prevention, personal and professional experiences of suicide ideation, suicide behaviour, death by suicide and perceived impact on police officers and staff.

## Analytic procedure

We utilised the reflexive thematic approach to data analysis which is participant-centred, data-driven and reflective (Braun and Clarke 2019). Consistent with this form of analysis, the data from interviews with participants from the three countries were combined, and analysis focused on investigating commonalities between datasets to address the research question. The coding development was undertaken by paired analysts from different disciplines to ensure interdisciplinary working. This interdisciplinary working recognised that to be applied and helpful in practice, the research needed to be police-focused as well as drawing on the range of skills offered by different research members. Our work was facilitated by two suicide prevention leads from the UK police forces partnering in the project and its dissemination. We used a dialogic and team approach which involves a series of collaborative discussions between members of the research team comprised of academics and police practitioners. During the mapping phase of collating conceptual codes, this dialogic approach enabled the research team to develop a final coding framework that utilised the insider knowledge of the police partners as well as the procedural knowledge of the academic researchers.

## Ethics

For all three countries, an ethical process with and via core gatekeepers was adhered to and approval provided, following overarching governance from the University of Leicester. Any research exploring suicide is ethically sensitive and requires a carefully monitored, iterative and responsive approach to the entire process of data collection, analysis and dissemination. The safeguarding of participants was supported by advice and guidance from specialist members of the team, including a psychiatrist, trauma-specialist clinical psychologist and the safeguarding teams within the police organisations. All participants were provided with an information sheet laying out the grounds of participation, boundaries for confidentiality breach, and anonymisation and protection of data. All participants provided written informed consent. The research team was provided with debriefing and supervision.

## Findings

Analysis of data generated four themes that related to the research question for this aspect of the study and included participants from all three countries. Thematic saturation was achieved in the sample from each country. These themes are represented in Table 1.

**Table 1.** Four themes generated.

**Theme one:** The context of working with suicide			
Frequency and number of suicides	Attending a suicide is a traumatic event	Culture of policing	
**Theme two**: The traumatic impact of working with suicide			
Trauma symptoms experienced	The cumulative impact of working with suicide	Long-lasting impact of attending certain suicides	Suicidal ideation amongst police
**Theme three**: The most difficult aspects of working with suicide			
Personal salience of certain suicide incidents	The challenges for police of communicating with families bereaved by suicide		
**Theme four**: Organisational opportunities for supporting work with suicide			
Stigma and career concerns as barriers to help-seeking	Suicide-specific support and training required		

### Theme one: the context of working with suicide

Although police work with many difficult incidents, including death, the data suggested that police officers and staff experienced all aspects of working with suicide as particularly traumatic. Within this theme, it was noted that working with those who were attempting to take their own life or had died by suicide was a significant part of the policing role. First, many participants highlighted the large numbers and frequency of suicide incidents. Second, participants listed the kinds of post-trauma symptoms they were experiencing after exposure to suicide. Third, it was also noted by participants that the current culture of policing practice tends to minimise the impact of working with suicide beneath a veneer of bravado.

#### Frequency and number of suicides

Police personnel are frontline responders to both death by suicide and attempted suicides, which according to the DSM-5 definition constitute a Criterion A traumatic event (American Psychiatric Association 2013). Notably, it was recognised by participants that dealing with traumatic incidents is becoming a larger proportion of the policing role.

*The number of traumatic incidents that we deal with is rising … And it will continue to rise because society’s going that way and there’s less cops*. (PO27-UK)

Indeed, in some places suicide response constitutes up to half of the policing role, in the sense that about 50% of their work in policing is occupied by working with deaths by suicide or with those attempting suicide.

*I’d say, maybe, 50% [*of workload*]*. (PO3-NZ)

The frequency of exposure to suicide for police officers and staff is high, and in some cases was reported to be a daily occurrence.

*I think you’ll be surprised by our extent of exposure to it. It’s every day.* (P1ConCom-UK)

*I have dealt with so many suicides and attempts, it's so many that I cannot remember the number.* (PO11-GY)

*Because it’s literally one after the other.* (PO30-UK)

An example of this was reported by participants in Guyana who specified peaks of suicide incidents in certain calendar months of the year.

*In region [name] … The suicide rate at [place] is very high. From September month end of last year, as well as November. They had 10 suicides in that period of time, and the age ranges from 16 to 45*. (PO7-GY)

#### Attending a suicide is a traumatic event

The DSM-5 PTSD diagnostic criteria, the Criterion A definition of a traumatic event includes exposure to ‘death, threatened death, actual or threatened serious injury’ (APA 2013, n.p). Although working with these events is an integral part of the policing role, participants reported that responding to death by suicide was qualitatively different.

*We go to a lot of sudden deaths and murders, whatever. But obviously suicides are a little bit different.* (PO32-UK)

In the data corpus, police responded to death by suicide accomplished by different means, including asphyxiation, electrocution, by firearm, hanging, cutting wrists, overdosing, carbon monoxide poisoning, setting oneself on fire and jumping from a height or in front of a train. Understandably, for police attending deaths by suicide by these means, the experience was graphic and often distressing.

*But the types of deaths on the railway certainly the suicides and particular, yeah, they can be particularly graphic and distressing*. (PO1-UK)

*I could tell you the colour of his tongue, of his face. I could tell you in vivid detail absolutely everything about that job.* (PO31-UK)

As the previous quote implies, being exposed to the bodies of people who have died by a means of suicide that has disfigured the corpse, is a vivid sensory experience. When exposed to a traumatic catalyst, the information processing part of the limbic system in the brain becomes hyper aware of the sights, sounds and smells associated with that incident (Kearney and Lanius 2022). As the following participant reported, the need to remove reminders of the sensory triggers can be overwhelming.

*And took me whole uniform off and walked back burnt to my boxers, ‘cause just that smell that, you know.* (P30PO-UK)

As the data in these two subsections illustrate, police participants consistently mentioned the number and frequency of suicides they attend, and the fact that attending suicide fulfils the APA definition of a traumatic incident. However, the police culture was reported to be a reason why the potential psychological impact on police officers and staff was minimised.

#### Culture of policing

Participants characterised the culture within the policing profession as one that expects employees to be able to embody a level of emotional detachment from the work. While personal resilience and emotional regulation is clearly a necessary aspect of professionalism, participants expressed concern that this expectation is over-emphasised in the police.

*You know, so yes, there is still that culture of we should be able to deal with anything. We should be able to see anything, do anything, um, and not have it affect us.* (PO27-UK)

*You know, that affected me as bad but, again, nobody … It’s just what cops do. It’s just what we do.* (PO23-UK)

*But we would have to stand strong and be able to lend the support and don't let our emotions or anything come into play.* (PO10-GY)

*You don't want them to see that giving is breaking down. So, what you try to do is just to show that you are strong. Emotionally, yeah.* (PO9-GY)

One of the primary aspects of police work is in dealing with the public. To work effectively with people, there is a need for police personnel to understand human emotions and to have a degree of awareness, empathy and relatability. Therefore, managing the balance between human connectedness and professional detachment from the more distressing aspects of the role, is understandably a challenging skill to navigate. Potentially, therefore some police present as being unaffected by the emotional valence of the work, and yet underneath they are impacted by what they see and hear.

*So, I don’t know if bravado’s the right word … What I mean is the cops who’s done it the most almost feel as if they’re been there and done that, so it doesn’t affect them as much, but it does.* (PO14-UK)

### Theme two: the traumatic impact of working with suicide

A significant theme was the impact on police personnel of working with suicide. There were four sub-themes created: first were data examples that narrated the kind of trauma symptoms experienced; second, participants noted the cumulative impact of working with multiple suicides over a period of time; third, memories of attending certain suicides were long-lasting, and fourth unfortunately, there were several participants who recounted that they had experienced suicidal ideation themselves.

#### Trauma symptoms experienced

In some interviews, participants explicitly stated they had experienced PTSD because of attending suicides. In other interviews, participants referred to trauma symptoms including the four DSM-5 clusters of intrusion, avoidance, alterations in cognition and mood, and alterations in arousal and reactivity (APA 2013).

*I think seven months after that year I was diagnosed with PTSD … there was male that was suicidal, and he ended his life whilst he was on the phone to me.* (P4Concom-UK)

*The main one was I was suffering from PTSD from the suicide.* (PO22-UK)

These examples are representative of some participants who directly related a PTSD diagnosis to their work with suicide. Other participants from all three countries made more indirect reference to being ‘traumatised’ by this kind of work.

*I was traumatised at first.* (PO1-GY)

‘*Cause you’re shell shocked yourself dealing with this.* (PO28-UK)

… *that vicarious trauma that police go through.* (PS4-NZ)

Regardless of whether formally diagnosed or not, many participants referred to experiencing symptoms of PTSD. In the following extracts participants described one of the symptoms, which is avoidance of reminders:

*I was just thinking, please don’t let him still be hanging … please don’t, because I do not want to see it.* (PO20*-*UK)

*I don’t want to go to another fatal. I do not want all that stress. ‘Cause I can’t cope with it myself.* (PO28-UK)

The following two extracts illustrate other trauma symptoms which are alterations in cognition and mood. Participants described changes in mood and difficulty sleeping.

*I don’t know why to this day, but I sat there and, you know, I cried. Until this day now I still do.* (PO2-UK)

*I went into a really sort of spiralling depression. Um, never really slept.* (PO30-UK)

One of the most characteristic symptoms resulting from experiencing trauma is intrusions and flashbacks, which were also mentioned by some participants.

*I don’t sleep afterwards for a while …  …  … So I have constant flashbacks to the body.* (PO9-NZ)

*Most times I would try to forget about the experience then a next case may pop up with the same scenario. And it's like you're reliving it again.* (PO8-GY)

#### The cumulative impact of working with suicide

For those new to the profession, the impact of working with suicide was reported to be shocking initially. Additionally, for those who had been in the profession for a while, they reported a cumulative impact whereby attending multiple suicides over several years began to have a more noticeable impact.

*I guess the cumulative effect does kind of affect me.* (PO3-NZ)

Although police officers and staff may have strategies to support their resilience, the ‘*weight*’ of attending multiple suicides over time was felt by many to become more and more burdensome.

*As opposed to it could accumulate and accumulate and build up. So, although it’s never affected me, I know of other officers who’ve done 30 or 40 and now they carry the weight of it.* (PO14-UK)

In describing the cumulative impact of working with suicide, participants argued that the ‘*build-up*’ of stressors gradually eroded their capacity to ‘*cope*’ effectively.

*I think it was some build-up of … Yeah, what it was, it was build-up of so many … . And that’s when it started to hit me.* (PO3-UK)

*Where I think there’s a lot of officers out there that can cope and dealing very well but it’s only a matter of time before it all comes crashing down.* (P4ConCom-UK)

*No matter how hard you try, you do carry it home.* (PO12-GY)

Problematically, the cumulation of engaging with this kind of work, means that police officers and staff find it increasingly difficult to separate the professional role from their personal home life.

#### Long-lasting impact of attending certain suicides

Participants reported that there were certain suicides that were particularly distressing. They stated that the memory of those particular events would be something that would stay with them for a long time, potentially forever.

*A lot of the suicides I’ve attended I’ll never forget.* (PO28-UK)

*One was where someone was pregnant at the time, couldn’t see a way out and that was, yeah, that still sits in my memory*. (P2ConCom-UK)

*I still think about it, and I think I probably will for the rest of my life, yeah.* (P5ConCom-UK)

The inability to move past the memory shows the degree of negative impact. The phrases participants used, ‘*I’ll never forget*’, ‘*still sits in my memory*’ and ‘*still think about it*’ indicate that the traumatic impact of what they experienced continued to haunt them, even many years later. Interestingly, two of these quotes are from staff in control and command, and even though they are one step removed from being in person at the scene of a suicide, they were still significantly affected.

#### Suicidal ideation amongst police

Sadly, one of the extreme consequences of the cumulative impact of working with suicide (alongside other work and personal factors), that participants reported was to consider suicide for themselves.

*And you go over the motorway bridge and I used to think about jumping off the bridge. And I knew it wasn’t right and these things gradually built.* (PO28-UK)

*In some of my lower points I suppose I had considered suicide myself.* (P6ConCom-UK)

These two examples demonstrate how the build-up of stressors had led both police officers on the ground and those in communication to be similarly impacted. In the following extract a participant who was currently working in the control room, had previously held another role, and described how they reached a point where the impact of the work left them ‘*ready to end my own life*’.

*It got to the point where I took so many suicidal people calls, a few rapes. I don’t think they bothered me … I think it was December 2018 I was at my worst, and I was ready to end my own life.* (P4ConCom-UK)

### Theme three: the most difficult aspects of working with suicide

As the previous themes introduced, police described how certain aspects of suicide response were more challenging than others. These challenges were first, when there was a personal resonance for the police officer in terms of the characteristics of the person who had died by suicide or the circumstances surrounding it. Second, the transition from attending to the body of the deceased, and then having to support the grieving family.

#### Personal salience of certain suicide incidents

For the most part, participants reported being able to maintain a degree of emotional separation in their work. However, there were particular incidents that participants reported as having greater personal salience than others, making it difficult to maintain a personal and professional segregation. This is-articulated by PO29 in the following example:

*It affects me more is when there’s something that’s quite um, relevant to me in it.* (PO29-UK)

When this participant stated that working with suicide was more difficult when it was *relevant to me*, personal resonance is implied. Personal resonance occurred when the suicide occurred in a familiar or local environment. For example, one participant mentioned the train station they commuted from every day.

*We had a child hit by a train back in December and it was at my home station. And I remember being on the radio channel and that was probably I’d say my worst day because I just felt sick … I just thought that one that’s a child I go to that station like … that’s my station every day.* (P5ConCom-UK)

When there was a specific aspect of the suicide that resonated with the participant’s personal circumstances, the meaning and impact was more profound. In the following extract, the child who died by suicide was the same age as the police officer’s son.

*And one of them which will always stand out to me is a 12-year-old lad who had an argument with his mum, um, and then he literally took a cord and hung himself in his bedroom …  … .And as I walked in, he looked like my son who’s twelve – exactly the height, hair, uh, the way he was facing away, and I just had to take a step back.* (PO26-UK)

*And part that resonated was the fact that I have a young son and this lad was wearing a t-shirt exactly the same as a t-shirt that my boy had that was probably his favourite t-shirt and he lived and died in* (P10-UK)

What these examples and the one that follows demonstrates is that the humanity of police personnel is integral to them, even in their professional role. Having empathy and compassion for others means that police officers and staff can work with the public in a more meaningful way. However, that same humanness can also mean that they are vulnerable to experiencing the emotional impact of working with the trauma of suicide. This was recognised by PO26 in the following example.

*Um and the trauma of, of the mum was just incredible. You know, she absolutely collapsed. And that’s really hard, you know. That was probably closest, you know, certainly I was welling up myself. Um, and I can remember going home … Um, I remember hugging my boy really tightly who was a bit younger, he’d have been about 11 … it’s so human. Um, and that, um, emotion, dealing with that, is so overpowering. It does eat into the side of doing a job and being human*. (PO26-UK)

The resonance of this example is the identification with another person, in this case, a parent identifying with the trauma of another parent bereaved by suicide. Recognising that their own child was a similar age provoked the need to physically connect with their own child when returning home. Although not all suicides have the same personal resonance, many participants reported they were emotionally challenging or overwhelming. A common sentiment was the combined traumas of attending the suicide scene and supporting the bereaved family was too much. Participants argued that it was therefore not appropriate to do this alone.

*I think it’s quite overwhelming for anyone, especially going into that scene … You shouldn’t be going to stuff like that on your own.* (PO25-UK)

*The challenges for police of communicating with families bereaved by suicide.*

#### The challenges for police working with bereaved families

In addition to managing the practical and legal aspects of the suicide incident, police officers are also required to deliver the news (i.e. the death message) to the next of kin. Participants reported this was the most difficult aspect of working with suicide.

*And that's often what comes out as being the worst thing, actually having to give the news.* (PS4-NZ)

Apart from having to inform family members that a loved one has died, an aspect of suicide that is qualitatively different from other means of death, is that family members typically feel they may be to blame in some way.

*They will look for someone to blame, right, it is classic, and it happens every single time because they will not accept … it’s normally because they deep down blame themselves, right? And they can’t deal with it.* (PO18-UK)

Participants described the fact that families were distraught and emotional was especially difficult to work with.

*And she was completely screaming her head off, she was crying, she was so over-wrought.* (PO23-UK)

At times, the family are the ones who have found the deceased person. In these situations, the two police tasks of dealing with the body and delivering news to the family become conflated into the same incident.

*Their reaction to that is the hardest thing I think for me. When I say about the family too like we’ve been to ones where the person’s hung themselves and they’re dead so they’re lying on the floor in the family home and there’s 20 family members just sitting around crying that’s sort of what I mean about the emotion.* (PO9-NZ)

*I think the trauma came from … from my point of view, comes from the family. If the family are there, then it ramps it up a little bit and it becomes harder to deal with.* (PO27-UK)

What participants reported was that although dealing with the body of the person who died by suicide was often a very challenging part of the role, having to communicate with the family was ‘*harder to deal with*’. Arguably, meeting a distressed family increases the personal salience of the incident. Additionally, participants indicated that the policies and training that they had been provided with was inadequate for preparing them for managing this part of the role.

*The thing that I don’t think the police do particularly well is the family … they don’t know how to talk to the family, and they don’t know how to communicate things and we’re quite robotic when we go to things like that.* (PO24-UK)

*I mean we’ve got a sudden death policy … and having read it, I’m not convinced that it tells you how you deal with the family.* (PO26-UK)

Communicating with distressed and bereaved relatives is a particular skill set that some participants proposed would be better addressed by a different person than the officer attending the suicide incident.

*Going out to a job where someone had done this and then having to go and do the bereavement message as well. This is something definitely forces need to look at that the officers shouldn’t be the same officer, it’s that simple.* (PO17-UK)

This theme has dealt with the most difficult aspects participants reported about working with suicide; incidents with personal resonance and working with bereaved families. As human beings, police personnel need to balance their humanity with professional detachment, however, the challenge of working with families is that it gives the incident personal and emotional meaning.

### Theme four: organisational opportunities for supporting work with suicide

Recently, there has been greater ambition of frontline organisations including the police to recognise and address some of the mental health and wellbeing needs experienced by the workforce. Pragmatically, supporting the wellbeing of staff reduces time taken on sick leave and potentially enhances productivity. Whilst acknowledging existing policies and practices in place to support police in this way, the data highlighted some areas where there could be opportunities for organisations to enhance their welfare offer. These areas are to recognise the stigma and concerns which may operate as a barrier to help-seeking, the introduction of more specific support around working with suicide, and the value of suicide-specific training.

#### Stigma and career concerns as barriers to help-seeking

Although there have been advances in mental health awareness, there remains a challenge of residual stigma regarding disclosing the need for mental health support. Police participants described how stigma reduced the likelihood of them seeking help.

*I think there is still a bit of a stigma around it’s a sign of weakness to ask for help*. (PO26-UK)

*Stigma. Stigma* [stops help-seeking]. *Pure and simple stigma.* (PO21-UK)

Even disclosing to colleagues that the work might be having an impact on wellbeing was described as difficult because of uncertainty about their reactions.

*I’ve got people that I work with, if I was really struggling, I don’t know how they’d react if I said no, I’m having a really bad day today … I’m really upset. How would they react?* (P5 ConCom-UK)

One of the fears participants reported in relation to help-seeking was that they might be seen as ‘soft’ or ‘weak’, which may negatively affect their potential for career progression.

*Even though they’re police officers, it’s still a policing environment, it’s difficult to admit and there’s a fear that if you say I’m not so comfortable with the work someone’s going to say, well you’ve got stress, you’re off, you know, well you can’t, you ain’t got a job anymore.* (PO1-UK)

*That suspicion, cynicism that it will get out, that it might not do your career any, any, any good. That, uh, people will think you’re soft.* (PO24-UK)

Participants acknowledged that despite the possible stigma of help-seeking, they nonetheless believed that therapy or counselling was beneficial.

*I believe the police should have a therapy session. I would call it because there are sometimes places where you need to let out all that you take in on a daily basis.* (PO4-GY)

In addition to identification of the need for psychological support within the police profession, some participants suggested that making this mandatory may be a way to counter stigma. As we have noted elsewhere, the routinisation of certain aspects of mental health conversations can be an effective normalisation strategy (see O’Reilly *et al*. 2016).

*I think if you make it mandatory there’s no stigma there, you’ve not chosen to do it, you’ve got to go because it’s part of the role, you make it mandatory, you’re going to engage.* (PO1-UK)

*If you do have some police who are having frustrations, depression, and suicidal thoughts, which we do have sometimes … They need to get somebody who is neutral, who is not a police officer.* (PO12-GY)

As the previous extract demonstrates, a further safeguard against potentially jeopardising career prospects by seeking help was the suggestion that therapy could be provided by professionals external to the organisation. While these suggestions may facilitate more culturally acceptable access to mental health support in general, participants also indicated that this would be particularly important for working with suicide.

#### Suicide-specific support and training needed

Participants recognised that working with death by suicide was qualitatively different from other causes of death. They believed that their training and support needs ought to be more specific to the nature of this work.

*Specially around suicide I don’t think it’s good. I don’t think the wrap around care and the wellness stuff we do for officers is specifically targeted for suicide.* (PO24-UK)

Although police officers and staff may be well trained in most areas of the work, participants in this study demonstrated that more specific support related to suicide was required.

*You’re never really taught anything you just wing it, don’t you?* (PO20-UK)

*I have never gone on any course or anything as it relates to suicide.* (PO7-GY)

*Because you don’t get a lot of training about this sort of thing at the college.* (PO1-NZ)

Considering the number of suicides that police officers attend in all three countries participating in this study, there is arguably a need for more specialised training in this area. This was a point that several of the participants made.

*Yeah, you should, you should be trained in suicide.* (PO22-UK)

*Yes, police need to be trained in suicide matters.* (PO1-GY)

*I think we should have classes to be edified more on suicide cases.* (PO2-GY)

The rationale for suicide-specific training was its connection to mental health. Participants felt that their role as police officers and staff was normatively focused on criminality and justice, and although responding to suicide might be perceived as on the periphery of their core business, the frequency meant it has become integral to their work.

*I think the major thing out of everything we’ve talked about for me is that there needs to be better training in what mental health issues and conditions are going to be encountered in the job and what to do with those. That training needs to be a lot better.* (PO9-UK)

*And also, I don’t know about yourself, but we never have any training in giving death messages.* (PO5-UK)

Notably, there were three core areas of training need that were discussed by participants. These were (i) dealing with the mental health crisis of suicidality, (ii) responding in situ to the scene of a person who has died by suicide and (iii) delivering the death message to the family.

## Discussion

It is important for police forces to have a healthy workforce, and health includes mental health. We recognise that parity of esteem between physical and mental health is a global challenge, and one that has political, cultural, community, health and social implications (Prince *et al*. 2007). Problematically, there is a global shortage of mental health professionals, and lower budgets for mental health provision, and yet mental health need is increasing (Bor *et al*. 2014; NHS digital 2018). Within this context, Human Resources and Wellbeing providers within police organisations are recognising the need for better mental health support. Indeed, they are already responding to this requirement within the resource constraints. However, our research indicates that police organisations would benefit from further attention to mental health promotion, the prevention of trauma and early intervention for mental health need. Indeed, in terms of prevention of suicide in police officers and staff, an organisational cultural shift that emphasises mental welfare to replace unhealthy coping strategies is needed (Krishnan *et al.* 2022).

More specifically, it is necessary to recognise that working with suicide is emotionally taxing, potentially traumatic, and impacts on the welfare of those staff. Managing the short and long-term impacts of working with suicide is important in the effort to reduce substance abuse, sickness or impaired performance. While our analysis highlighted some improvements regarding attention to and support for mental health and wellbeing in the police, there remain areas of work with suicide that still require resourcing. Organisational improvements operate alongside wider societal, cultural and social changes in relation to how mental health is viewed and treated, and in relation to help-seeking behaviours. These wider influences need to be accounted for in any strategic and operational work. Furthermore, it is necessary to routinely listen and engage with police in promoting organisational change.

Notwithstanding the limitations that participants volunteering may be those who have a specific interest in the topic area, there were important findings from the study. The clear message demonstrated through the themes, was that police are human and experience the range of human emotions associated with working with the trauma of others. It was argued by participants that the nature of the role is already stressful, making those working in the field vulnerable to mental health need. In relation to the DSM-5 PTSD Criterion A, working with suicide constitutes exposure to a traumatic event, and some participants articulated symptoms including avoidance, hypervigilance, negative mood and thoughts (APA 2013). Death by suicide was identified by participants as being qualitatively different from other forms of death, and often more difficult to emotionally process. It was also noted that there was a cumulative emotional impact from the increased number of suicides police are expected to attend. They acknowledged there were certain aspects of working with suicide that made this part of the role especially difficult, including informing bereaved families, the sensory experience of the death scene, and personal identifiers or thought processes that exacerbated the encounter. These challenges were argued to be hindered further by the lack of preparation for working with suicide when entering the career, and limited suicide-specific training. This is despite research showing that it would be beneficial for them to receive suicide-specific training (Cerel *et al.* 2019). Interestingly, our findings also indicated that staff in the control and command room were considerably affected by responding to suicide and therefore also need the same training and support as frontline officers.

Modern police forces have put in place support structures for staff wellbeing and mental health. There are resources and help available in many police organisations designed to support those exposed to trauma, and the participants acknowledged that their organisations did have a support system in place for mental health and wellbeing. However, while these are incredibly beneficial and were typically positively appraised by participants in the study, they were ultimately argued to be insufficient in various ways for working with suicide specifically. Participants believed that the welfare offer in place generally was not oriented toward working with suicide and did not sufficiently acknowledge its uniqueness as a trauma. Accessing support typically relied on personal disclosures to managers who were not always equipped or resourced and risked having negative career consequences. Furthermore, the welfare supports available operate within an organisational culture of wider expectations of resilience, a perceived lack of support, limited resources, stigma and resistance to help-seeking. Consequently, these broader systemic challenges functioned as barriers to help-seeking in ways that reduced engagement with, and the effectiveness of, the wellbeing resources available. Founded in the voices of the participants, their concerns, experiences, ideas and challenges, we argue therefore that there are ways in which police organisations can support their officers and staff working with suicide and make several recommendations. In so doing, we acknowledge that these recommendations are based on this single qualitative study, which while having a large sample size in qualitative terms and spanning three countries, does need further research work to evaluate the benefit of implementing these in practice. This research has focused on identifying thematic similarities between the experiences of police in the United Kingdom, Guyana and New Zealand, which has the benefit of providing an initial exploration of the important issues at stake for policing internationally. However, further research is also recommended to explore cultural, social and political differences between countries and a more comprehensive comparative study across more countries is clearly needed.

### Training

It is widely recognised that working with suicide is a central role for the police, and the World Health Organisation (2009) acknowledged that police should be trained to identify risks of suicide. Evident from the participants’ narratives was a feeling of lack of preparedness to work with suicide, a limited knowledge of suicide at the outset of their career, and feelings of concern around competencies to work with bereaved families. We argue that police organisations could provide additional training sessions specific to working with suicide for new recruits alongside ‘top-up’ training for more experienced officers. Training in suicide awareness, suicide communication and mental health management could all be beneficial. More specifically, there are two aspects of this training that are focussed on public facing activity which are:

1. Skills for the job of working with suicidal persons.
2. Skills for communicating with the bereaved family.

In addition, a further aspect of training would focus on skills related to self-care given the cumulative physiological burden of work with people with suicidal intention and with families bereaved by suicide.

In so doing, organisations have a responsibility to provide adequate resources and space for officers to fully embrace the training as part of natural workload allocations and not allow it to be an optional ‘tag on’ between other tasks. Any training needs to be tailored to the occupational group, cultural context and geographical location of the force. Research indicates that training in suicide and related matters is typically well-received by officers (Marzano *et al.* 2016). Additionally, many police organisations have a specialised Suicide Prevention Lead (or similar), and this role could be valuable in designing education for police officers and staff. Additional training on communication with families, especially delivering the ‘death message’ would be beneficial.

### Mental health care

Participants reported that working with suicide is qualitatively different from working with other forms of death. It is arguably necessary therefore that police organisations recognise this at a policy and procedural level, making organisational adjustments to suicide pre- and post-incident training and support for officers. Officers require a greater awareness of pre-, peri- and post-incident self-care. Furthermore, we suggest that the end of shift debrief does not occur in isolation, and a follow-up supportive conversation 48 h later is offered (Mitchell and Everly 1997).

The participants reported a high frequency in working with suicide and felt that they sometimes were unable to cope with the volume, especially ones that were traumatic and difficult. Where resources allow, police organisations could create a threshold of the number of suicides attended or worked with during a specified period. Furthermore, where possible organisations could consider not sending officers alone to sudden deaths. It is therefore important that those occupying managerial positions have the competencies and skills necessary to promote such responses and are equipped with the information they need to support their staff.

### Dealing with trauma

According to the DSM-5 PTSD Criterion A attending a scene where a person has died by suicide would constitute a traumatic event. Although many police officers and staff do not go on to develop full PTSD symptoms, identifying early signs of the impact of trauma is important to reduce the risk of poorer long-term outcomes. While detachment and avoidance are common short-term coping strategies, it is important for long-term mental health that officers and staff learn healthy stress regulation strategies.

Police organisations have an essential role to play in helping officers and staff manage trauma. They could benefit from following a trauma-responsive protocol that acknowledges the cumulative impact of working with suicide. The cumulative impact of attending many suicides during a career can negatively affect officer’s perceptions of life and death, and their ways of coping (Galloucis *et al.* 2000). While potentially expensive, regular clinical supervision could have the benefit of early identification of trauma symptoms and thus could be an effective prevention technique. Additionally, supervision can facilitate effective self-management of stress thus optimising executive functioning and potentially reducing the likelihood of sickness, performance errors and suicidal ideation. This could be facilitated using specific tools, like the Secondary Traumatic Stress Scale, which identifies elements of traumatic stress (Hurrell *et al.* 2018).

### Police culture and organisational change

Implementation of recommendations in a successful way that supports help-seeking and reduces trauma relies on the culture of the organisation. We argue that police organisations would benefit from a culture that recognises the traumatic impact of working with suicide, and introduce a *suicide trauma-responsive culture*, that recognises trauma symptoms are a normal reaction to an abnormal event. In each of the countries studied, the emotional labour of policing work was evident. However, in undertaking the work, there is a need for a certain amount of emotional detachment to ensure professionalism in conducting the institutional business. Research shows that emotional suppression in policing goes beyond interactions with the public and extends to peer and family relationships (Lennie *et al.* 2020). Yet, the humanness of the police officer or staff needs to be acknowledged and recognised in all police organisations in all three countries. Officers and staff can be actively encouraged to monitor their own mental health and wellbeing and seek support where needed without fear of negative consequences. This needs to be embedded in discourse, action and policy and needs to come from the top. Senior leaders could actively show that seeking support for their mental health will not impact career progression.

## Conclusion

In conclusion, we argue that there are different levels of support that need to be operationalised for improving the care of police officers and staff involved in responding to suicide. First, is the level of self-care, as those working for the police have some autonomy and agency to manage their own mental health and have some responsibility to seek-help. Second, is the divisional level of care within a department, and the line manager needs to be responsive and alert to red flags that indicate implementation of better systems to support mental health and wellbeing. Third, is the organisational level, whereby a cultural shift is needed, sensible organisational policies that recognise the impact of working with suicide as unique and addressing resource challenges and workload issues. Fourth, is the systemic level, as police officers and organisations do not operate within a vacuum from society, and challenges such as stigma, gendered social norms and public perceptions and expectations will influence the way in which the police manage their work with and responses to suicide. Finally, at the political level, governments have a responsibility toward those who represent public sector services, including wider suicide prevention policies, investment in research into suicide, and sufficient funding for mental health support for personnel in public services. In closing our argument therefore, we advocate that more attention is paid to frontline professionals working with suicide, as they grapple every day with the emotion, trauma and consequences of their role.
